# Discontinuation of biosimilar infliximab in Japanese patients with rheumatoid arthritis achieving sustained clinical remission or low disease activity during the IFX-SIRIUS STUDY I (the IFX-SIRIUS STUDY II): Study protocol for an interventional, multicenter, open-label, single-arm clinical trial with clinical, ultrasound and biomarker assessments

**DOI:** 10.1097/MD.0000000000021480

**Published:** 2020-08-07

**Authors:** Toshimasa Shimizu, Shin-Ya Kawashiri, Shuntaro Sato, Shimpei Morimoto, Shuri Minoda, Yurika Kawazoe, Shohei Kuroda, Shigeki Tashiro, Remi Sumiyoshi, Naoki Hosogaya, Hiroshi Yamamoto, Atsushi Kawakami

**Affiliations:** a Clinical Research Center, Nagasaki University Hospital; b Departments of Immunology and Rheumatology; c Community Medicine and, Division of Advanced Preventive Medical Sciences, Nagasaki University Graduate School of Biomedical Sciences; d Innovation Platform & Office for Precision Medicine, Nagasaki University Graduate School of Biomedical Sciences, Nagasaki, Japan.

**Keywords:** biomarker, biosimilar, CT-P13, musculoskeletal ultrasound (MSUS), rheumatoid arthritis (RA)

## Abstract

**Background:**

The introduction of biological disease-modifying anti-rheumatic drugs into clinical practice has dramatically improved the clinical outcomes of individuals with rheumatoid arthritis (RA). We are conducting the IFX-SIRIUS STUDY I that evaluates whether switching from originator infliximab (IFX) to its biosimilar, CT-P13, is not inferior in maintaining nonclinical relapse to continue treatment with originator IFX in patients with RA achieving clinical remission. It is the next great issue whether disease activity can be maintained in good condition after discontinuation of CT-P13 because no evidence is available regarding the clinical value of discontinuing biosimilars in patients with RA. Thus, we will evaluate whether a condition without clinical relapse will be maintained after discontinuation of CT-P13 in patients with RA, achieving clinical remission or low disease activity during the IFX-SIRIUS STUDY I.

**Methods/design:**

This study is an interventional, multicenter, open-label, single-arm clinical trial with a 48-week follow-up. Patients with RA who are treated with CT-P13 and sustained nonclinical relapse during the IFX-SIRIUS STUDY I will be included. Patients will discontinue CT-P13 after the study period of the IFX-SIRIUS STUDY I. We will evaluate disease activity by clinical disease activity indices and musculoskeletal ultrasound (MSUS). The primary endpoint is the proportion of patients who do not have clinical relapse during the study period. Important secondary endpoints are the changes from the baseline of the MSUS scores. We will also comprehensively analyze the serum levels of multiple biomarkers, such as cytokines and chemokines. In addition, if a clinical relapse occurs in patients after the discontinuation of CT-P13, we will evaluate the effectiveness and safety of restarting CT-P13.

**Discussion:**

The study results are expected to show the clinical benefit of the discontinuation of CT-P13 and effectiveness and safety of restarting CT-P13 after clinical relapse. The strength of this study is to prospectively evaluate the therapeutic effectiveness by not only clinical disease activity indices but also standardized MSUS findings in multiple centers. We will explore whether parameters at baseline can predict a nonclinical relapse after the discontinuation of CT-P13 by integrating multilateral assessments, that is, patient's characteristics, clinical disease activity indices, MSUS findings, and serum biomarkers.

**Trial registration:**

This study was registered in the Japan Registry of Clinical Trials (https://jrct.niph.go.jp) on April 20, 2020 as jRCTs071200007.

## 1 Introduction

Rheumatoid arthritis (RA) is a chronic, systemic inflammatory disease that primarily involves synovial joints.^[1]^ The uncontrolled disease activity of RA may lead to joint destruction and deformity, causing impaired quality of life. Thus, tight control of disease activity by the treat-to-target strategy is recommended to prevent joint destruction.^[2]^ Advances in RA treatment, such as the use of biological originator disease-modifying anti-rheumatic drugs (boDMARDs) and biosimilar DMARDs (bsDMARDs), have provided better clinical outcomes, including the achievement of low disease activity or clinical remission with RA.

We are conducting IFX-SIRIUS STUDY I (jRCTs071190030) that evaluates whether switching from originator infliximab (IFX) to CT-P13, a biosimilar of originator IFX developed by Celltrion (Incheon, South Korea), is not inferior in maintaining nonclinical relapse to continue treatment with originator IFX in patients with RA achieving clinical remission.^[3]^ Switching from boDMARDs to bsDMARDs is expected to reduce patients’ economic burden and improve medical insurance finances; however, the continuation of bsDMARDs is still costly. Therefore, it is the next great issue whether disease activity can be maintained in good condition after discontinuation of CT-P13 in patients with RA.

Several previous reports showed the clinical benefit of the discontinuation of boDMARDs in patients with RA.^[4–6]^ Thus, we expect that patients with RA will be able to maintain good outcomes after the discontinuation of CT-P13. However, there is no evidence on the optimal approaches of discontinuation of bsDMARDs, such as CT-P13. Hence, it is desirable to investigate the clinical benefit of the discontinuation of CT-P13 in patients with RA, maintaining clinical remission or low disease activity by treatment with CT-P13. We will also evaluate disease activity using not only clinical disease activity indices but also musculoskeletal ultrasound (MSUS) to accurately assess inflammation at the joint level in this study, continuing from the IFX-SIRIUS STUDY I. In addition, we will comprehensively analyze the serum level of multiple biomarkers, such as cytokines and chemokines, and explore whether patients’ parameters at baseline can predict a nonclinical relapse after discontinuation of CT-P13 by integrating multilateral assessments, including patient's characteristics, clinical disease activity indices, MSUS findings, and serum biomarkers.

Furthermore, we expect that a certain proportion of patients with RA will have clinical relapse after discontinuation of CT-P13; thus, we will also evaluate the effectiveness and safety of CT-P13 retreatment in patients with RA who have clinical relapse.

We named this clinical trial IFX-SIRIUS STUDY II because this is a continuation trial of IFX-SIRIUS STUDY I. Herein, we describe the final study protocol (version 1.0; March 26, 2020).

## 2 Objectives

### 2.1 Primary objective

The principal objective of the study is to determine the proportion of patients who maintained nonclinical relapse after discontinuation of CT-P13 in patients with RA treated with CT-P13 during the period of IFX-SIRIUS STUDY I.

### 2.2 Secondary objectives

We will assess disease activity by MSUS after the discontinuation of CT-P13. We will explore whether patients’ baseline parameters, including clinical disease activity indices, MSUS scores, and serum biomarkers, can predict a nonclinical relapse after the discontinuation of CT-P13. Additionally, we will assess the effectiveness and safety of restarting CT-P13 in patients with clinical relapse during the study period.

## 3 Methods/design

### 3.1 Study design

**Figure 1:**
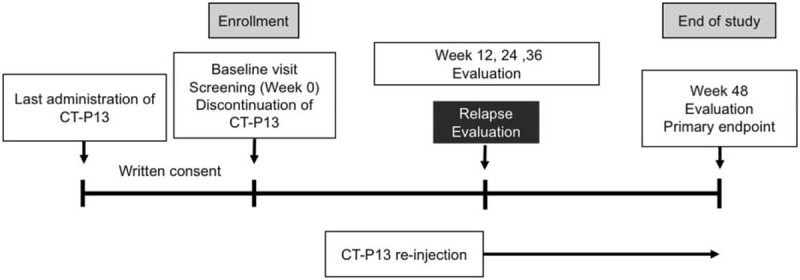
Study design.

The study design is in accordance with the Standard Protocol Items: Recommendations for Interventional Trials and Consolidated Standards of Reporting Trials 2010 guidelines^[7,8]^ (Additional File 1). The study is a prospective, open-label, single-arm, and interventional clinical trial. It will be conducted at the following 20 centers: Nagasaki University Hospital, Asahi General Hospital, National Hospital Organization Chiba-East Hospital, Eiraku Clinic, Hamanomachi Hospital, Japanese Red Cross Nagasaki Genbaku Hospital, Kagawa University Hospital, University of Miyazaki Hospital, Nagasaki Kita Hospital, Osaka City University Hospital, Osaka Medical College Hospital, Kindai University Hospital, Tobata General Hospital, Fukushima Medical University, Sagawa Akira Rheumatology Clinic, Sasebo Chuo Hospital, Sasebo City General Hospital, Shiminnomori Hospital, Utazu Hospital, and Yoshitama Clinic for Rheumatic Diseases. Patients with RA who have nonclinical relapse during the period of IFX-SIRIUS STUDY I will be assigned to discontinue CT-P13. The duration of the intervention is 48 weeks. The study design is summarized in Figure 1.

### 3.2 Approvals

The study was approved by the certified review board (CRB) of Nagasaki University (CRB approval no.: CRB20-003). The study was registered in the Japan Registry of Clinical Trials (https://jrct.niph.go.jp) as jRCTs071200007. We will conduct the study in accordance with the principles of the Declaration of Helsinki and Clinical Trials Act (Act No. 16 of April 14, 2017), Act on the Protection of Personal Information and related regulatory notifications, and this clinical study protocol. Participants will be provided with an explanation regarding the study by their treating rheumatologist and asked to voluntarily sign an informed consent form before participation.

### 3.3 Participants

#### 3.3.1 Inclusion criteria

Patients must meet all of the following requirements to be considered for entry into the study:

(1) treatment with CT-P13 and nonclinical relapse during the period of IFX-SIRIUS STUDY I and
(2) ability and willingness to provide written informed consent and comply with the requirements of the study protocol.

#### 3.3.2 Exclusion criteria

The exclusion criteria are as follows:

(1) history of infusion reaction due to CT-P13 that required medication;
(2) treatment with a corticosteroid or anti-rheumatic drug and change of dose after the period of IFX-SIRIUS STUDY I prior to the baseline visit;
(3) use of a prohibited drug or therapy after the period of IFX-SIRIUS STUDY I prior to the baseline visit;
(4) detection of a pregnancy after the period of IFX-SIRIUS STUDY I prior to the baseline visit;
(5) current pregnancy, breastfeeding, or noncompliance with a medically approved contraceptive regimen during the study period; and
(6) inappropriateness for inclusion in this study as determined by the investigator.

#### 3.3.3 Sample size

As a consequence of the inclusion criteria

(1) (section 3.3.1), the sample size for this study will be ≤ 80, that is, the sample size for the preceding study (IFX-SIRIUS STUDY I).^[3]^ We assumed that approximately 70% of 80 participants in the preceding study would meet the inclusion criteria of the present study; hence, the sample size of this study is expected to be approximately 50 to 60.

#### 3.3.4 Intervention

Patients will discontinue intravenous CT-P13 throughout the study period. If a clinical relapse occurs in patients after CT-P13 discontinuation, CT-P13 will be re-administered at 3 mg/kg at 0, 2, and 6 weeks. The same dose will be administered every 8 weeks after 14 weeks.

All patients must continue to receive the same doses of methotrexate and oral corticosteroid that they were receiving before the discontinuation of CT-P13 throughout the study period. During the study period, the following treatments are prohibited: administration of a bDMARD or JAK inhibitor, concomitant use of an immunosuppressant (azathioprine, cyclophosphamide, cyclosporine) or oral corticosteroids equivalent to > 10 mg/day of prednisolone, intra-articular corticosteroid injections at joints, and nonsteroidal anti-inflammatory drug (NSAID) suppositories. During the study period, the dose of any NSAID can be modified within the range of its approved doses in Japan.

#### 3.3.5 Patient discontinuation criteria

A patient may be prematurely withdrawn from the study for the following reasons:

- Continuing participation is inadvisable due to adverse event(s) (AEs).
- The patient asks to leave the trial.
- In the principal investigator's discretion, continuation in the trial would be detrimental to the patient's well-being.

### 3.4 Outcome measurements

Study visits will be conducted at baseline and 12, 24, 36, and 48 weeks after the discontinuation of CT-P13. If a clinical relapse occurs, the patient will visit for relapse evaluation. The assessments are presented in Table 1. Clinical physicians will be blinded to joint assessments by MSUS.

**Table 1:**
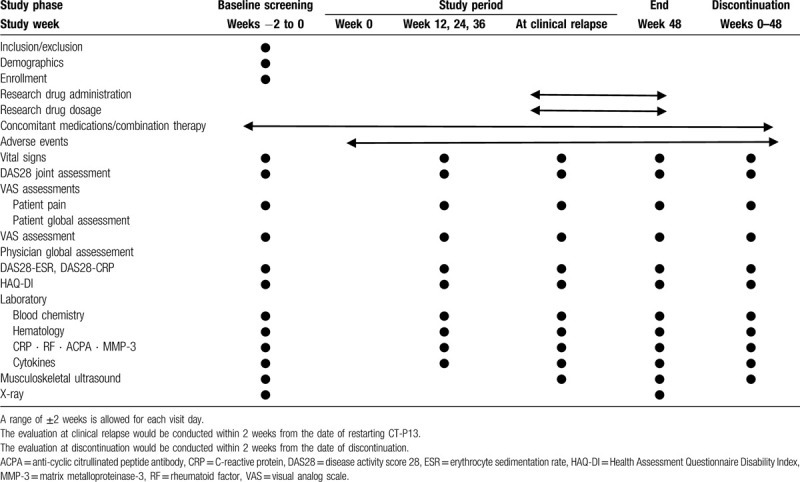
Treatment schedule and outcome measures.

#### 3.4.1 Clinical disease activity

Clinical disease activity will be evaluated by each attending physician (Japan College of Rheumatology [JCR]-certified rheumatologists) based on the values of the Disease Activity Score-28 (DAS28)-erythrocyte sedimentation rate (ESR) and DAS28-C reactive protein (CRP) level.^[9]^ At each visit, 28 joints, including the bilateral glenohumeral, elbow, wrist, metacarpophalangeal (MCP), interphalangeal (IP), proximal interphalangeal (PIP) of the hand, and knee joints, will be assessed for tenderness and swelling. Each patient's global assessment (PtGA) and evaluator's global assessment will be established on a 0–100-mm visual analog scale. The patient's functional assessment will be evaluated by the health assessment questionnaire-disability index (HAQ-DI).^[10]^

#### 3.4.2 MSUS assessments

Participants will undergo imaging by MSUS at baseline, week 48, and clinical relapse. The MSUS examination of each patient will be performed by one of the JCR-certified sonographers. A systematic multiplanar grayscale (GS) and power Doppler (PD) examination of each patient's joints will be performed using a multifrequency linear transducer (12–24 MHz). PD will be used depending on which Doppler modality is the most sensitive on the individual machines. The Doppler settings will be adjusted at each hospital according to published recommendations.^[11]^ There will be no change in the MSUS settings during the study and no upgrading of software.

Articular synovitis will be assessed by MSUS at dorsal views of 22 joints: bilateral wrist joints, 1st–5th MCP joints, IP joints, and 2nd–5th PIP joints. Each joint is scored for GS and PD on a scale of 0 to 3 in a semiquantitative manner. The sum of the GS or PD scores is considered the total GS or PD score, respectively. We will also assess the Outcome Measures in Rheumatology (OMERACT)-European League Against Rheumatism (EULAR) combined PDUS score (i.e., the combined PD score) and Global OMERACT-EULAR Synovitis Score.^[12,13]^ The combined PD score is combined with synovial hypertrophy shown by GS and PD.^[12,13]^

#### 3.4.3 X-ray imaging

X-ray imaging of bilateral hands (posteroanterior view) and feet (anteroposterior view) will be conducted. Joint damage progression will be evaluated based on the van der Heijde-modified total Sharp score (vdH-mTSS) method including 16 areas in each hand for erosions and 15 for joint-space narrowing.^[14]^

#### 3.4.4 Biomarker measurements

The patient's serum concentrations of the following biomarkers will be measured: Rheumatoid factor (RF) will be measured using latex agglutination turbidimetric immunoassay (LATIA) (LZ test “Eiken” RF). Anti-cyclic citrullinated peptide antibodies will be measured using chemiluminescent immunoassay (STACIA MEBLux test CCP). Matrix metalloproteinase-3 (MMP-3) will be measured using latex turbidimetric immunoassay (Panaclear MMP-3 “Latex”). Multiplex cytokine/chemokine bead assays will be performed using diluted serum supernatants and MILLIPLEX MAP Human Cytokine/Chemokine Magnetic Bead Panel (Merck Millipore) – Bio-Plex Pro Human Cytokine Assays (Bio-Rad) analyzed with a Bio-Plex MAGPIX Multiplex Reader (Bio-Rad) according to the manufacturer's instructions.

The cytokines/chemokines that are measured by the bead panel include interleukin (IL)-1α, IL-1β, IL-1 receptor antagonist, IL-2, IL-3, IL-4, IL-5, IL-6, IL-7, IL-8, IL-9, IL-10, IL-12 (p40), IL-12 (p70), IL-13, IL-15, IL-17, IL-18, interferon-gamma (IFN-γ), IFN-α2, CXCL1 (growth-related oncogene [GRO]), granulocyte-macrophage colony-stimulating factor (GM-CSF), granulocyte CSF (G-CSF), CX3CL1 (fractalkine), flt-3 ligand, fibroblast growth factor-2, eotaxin, epidermal growth factor, soluble CD40 ligand, vascular endothelial growth factor, tumor necrosis factor (TNF)-β, TNF-α, transforming growth factor-α, CCL4 (macrophage inflammatory protein [MIP]-1β), CCL3 (MIP-1α), CCL22 (macrophage-derived chemokine), CCL7 (monocyte chemotactic protein [MCP]-3), CCL2 (MCP-1), CXCL10 (IFN-γ-inducible protein-10), vascular cell adhesion molecule-1, and intercellular adhesion molecule-1. The serum IL-6 and TNF-α levels will be measured using specific enzyme-linked immunosorbent assay kits (R&D Systems).

### 3.5 Study endpoints

#### 3.5.1 Primary endpoint

The primary endpoint is the proportion of patients who had clinical relapse during the period from baseline to week 48. Clinical relapse is defined as

(1) a change from the baseline value in the DAS28-ESR (ΔDAS28-ESR) ≥1.2 or DAS28-ESR ≥3.2 and
(2) an increase in the DAS28-ESR value only due to increased disease activity of RA.

#### 3.5.2 Secondary endpoints

The secondary endpoints of this study are as follows:

(1) changes in the total PD and GS scores and combined PD score from baseline to week 48 and
(2) changes in the DAS28-ESR and DAS28-CRP values from baseline to weeks 12, 24, 36, and 48.

If patients had clinical relapse, the following endpoints are added:

(1) changes in the total PD and GS scores and combined PD score from relapse evaluation to week 48 and
(2) changes in the DAS28-ESR and DAS28-CRP values from relapse evaluation to each study visit after relapse evaluation.

#### 3.5.3 Exploratory endpoints

For further research, we will assess the following:

1. change in vdH-mTSS from baseline to week 48
2. change in the HAQ-DI data from baseline to weeks 12, 24, 36, and 48
3. changes in the serum levels of biomarkers from baseline to weeks 12, 24, 36, and 48
4. DAS28-ESR and DAS28-CRP values at baseline and weeks 12, 24, 36, and 48
5. total PD and GS scores and combined PD score at baseline and week 48
6. vdH-mTSS at baseline and week 48
7. HAQ-DI at baseline and weeks 12, 24, 36, and 48
8. serum levels of biomarkers at baseline and weeks 12, 24, 36, and 48.

If patients had clinical relapse, we will also assess the following:

1. change in the HAQ-DI data from relapse evaluation to each study visit after relapse evaluation
2. changes in the serum levels of biomarkers from relapse evaluation to each study visit after relapse evaluation
3. DAS28-ESR and DAS28-CRP values at relapse evaluation
4. total PD and GS scores and combined PD score at relapse evaluation
5. HAQ-DI at baseline at relapse evaluation
6. serum levels of biomarkers at relapse evaluation.

### 3.6 Adverse events

All AEs that occur between the baseline visit and end of week 48 will be recorded. If necessary, the investigators will administer treatments. A serious AE (SAE) is defined as any adverse reaction resulting in any of the following outcomes: life-threatening condition or death; condition that requires inpatient hospitalization or prolongation of existing hospitalization; and condition threatening to cause disability or disability, congenital anomaly, or birth defect. All SAEs will be documented in the medical records and reported to the CRB by the responsible investigator in accordance with Japanese regulations.

### 3.7 Data collection and management

Appropriate and authorized persons (investigators, clinical trial physicians, and clinical trial collaborators) will prepare a case report form (CRF) for each patient. All data recorded in the CRF must be consistent with the original material. According to the schedule presented in Table 1, the investigator will collect data at each patient visit during the study. The investigators will be provided access to an online, web-based, electronic data-capture system. Only the investigator and research supporter will be able to enter and modify data in the electronic CRF (e-CRF). All study findings and documents will be regarded as confidential. Patients will be identified on the e-CRF by each anonymous number, not by name. The confidentiality of the documents that identify the patient must be maintained by the investigator so that the anonymity of the participants is ensured. During the study, a sponsor-investigator will perform regular site visits to review protocol compliance, conduct source data verification, assess drug accountability and management, and ensure that the study is being conducted according to pertinent regulatory and protocol requirements.

### 3.8 Statistical analysis method

The primary analysis is planned to estimate the 95% confidence interval of the proportion that is stated as the primary endpoint (section 3.5.1) via Wilson score interval.^[15]^ The primary endpoint is evaluated in a set of subjects with either a result from the primary endpoint evaluation at week 48 or an observed clinical relapse during the observation period. To support clinical interpretation of the result from primary analysis, subject-wise time courses of DAS28-ESR and DAS28-CRP scores are plotted. Moreover, the cumulative number of events is plotted as a step function of time. The distribution of the changes in the total PD and GS scores and the combined PD scores is summarized as histograms.

Other statistical analyses are planned to be exploratively conducted on the relationships between measuring results obtained as exploratory outcomes, which are detailed above in section 3.5.3.

## 4 Discussion

The main purpose of this clinical trial is to evaluate whether a condition without clinical relapse will be maintained after the discontinuation of CT-P13 in patients with RA, achieving clinical remission or low disease activity during IFX-SIRIUS STUDY I. Although the introduction of biologics into clinical practice has dramatically improved the outcomes in RA,^[16]^ the currently available biologics are expensive, which has led to restricted treatment access in patients with RA.^[17]^ Switching from originator infliximab to CT-P13 will play an important role in cost savings and health gains for patients with RA. However, long-term continuation of CT-P13 is still costly.

Several studies have demonstrated the clinical benefits of the discontinuation of boDMARDs in patients with RA. The RRR study showed that 55% of patients with RA could maintain nonclinical relapse 1 year after the discontinuation of IFX.^[4]^ The HONOR study showed that 62% of patients with RA could maintain nonclinical relapse 1 year after the discontinuation of adalimumab.^[5]^ However, no clinical study has investigated the clinical benefit of the discontinuation of bsDMARDs in patients with RA. We will evaluate changes in disease activity after the discontinuation of CT-P13 in patients with RA who sustained a good clinical condition.

Moreover, re-administration of CT-P13 is concerned with the possibility of appearance of drug antibodies and infusion reaction, which affect the effectiveness and safety of CT-P13. Therefore, it is important to evaluate the effectiveness and safety of the re-administration of CT-P13 in patients with RA who had clinical relapse after the discontinuation of CTP-13.

The strength of this study is that it prospectively evaluates the therapeutic effectiveness using not only clinical disease activity indices but also standardized MSUS findings in multiple centers. We will evaluate accurately and objectively disease activity at the joint level. We will also analyze comprehensively the serum levels of multiple biomarkers, such as cytokines and chemokines. We will explore whether parameters at baseline can predict a nonclinical relapse after the discontinuation of CT-P13 by integrating multilateral assessments (i.e., patient's characteristics, clinical disease activity indices, MSUS findings, and serum biomarkers). It would be cost effective if we could predict from the results of two consecutive studies, IFX-SIRIUS STUDY I and II, that patients on stable treatment with originator IFX could switch to CT-P13 without relapse and could then discontinue CT-P13 without relapse before switching.

## Acknowledgments

We thank our colleagues and staff at the Rheumatology Department of Nagasaki University Hospital for their support.

## Author contributions

**Conceptualization**: SY. Kawashiri, T. Shimizu, H. Yamamoto, A. Kawakami

**Formal analysis**: SY. Kawashiri, T. Shimizu, S. Sato, S. Morimoto

**Investigation**: SY. Kawashiri, T. Shimizu, R. Sumiyoshi, A. Kawakami

**Methodology**: SY. Kawashiri, T. Shimizu, S. Sato, S. Morimoto, N. Hosogaya, H. Yamamoto, A. Kawakami

**Project administration**: SY. Kawashiri, T. Shimizu, S. Minoda, Y. Kawazoe

**Writing – original draft**: T. Shimizu, SY. Kawashiri

**Writing – review & editing**: T. Shimizu, SY. Kawashiri, S. Sato, S. Morimoto, Y. Kawazoe, S. Kuroda, S. Tashiro, A. Kawakami.
